# St. Thomas's Hospital

**Published:** 1831

**Authors:** 


### ST. THOMAS'S HOSPITAL.

### VENEREAL DISEASES.

As opinions have differed so widely for many years upon the important subject of diseases produced by sexual intercourse, and such variance still exists among authors and practitioners of the present day on almost every point connected with their origin, mode of progress, the nature of the different appearances which they assume, and more particularly in the methods of treatment, we have thought a few columns would be well filled in selecting some cases for publication from the venereal wards of this institution, which admit of a greater number of inmates than those of any other general hospital in the metropolis. Examples of the most common form in which venereal diseases daily shew themselves are here presented, and from the facts themselves, which are of more interest and value than any observations we can offer, may be derived the sentiments and practice of the surgeons of this hospital.

### I. GONORRHŒA AND ITS CONSEQUENCES.

CASE 1.—*Simple Gonorrhœa.* John Collins, æt. 23, was received into Job's Ward, Dec. 30th, 1830. Has had a gonorrhœal discharge since Saturday last, the 25th, which was the fourth day after connexion. There is slight redness at the orifice of the urethra, but no inflammation of the prepuce nor enlargement of the penis. Has some ardor urinæ, which he says is less than it has been, and trifling chordee at night. No sore upon the genitals, nor recent glandular swelling is to be observed. He suffered from the same complaint nearly a year ago, when one of the inguinal glands became inflamed, which has never entirely subsided, and there now remains some enlargement in the groin—hard, but not tender to the touch.

Ordered by Mr. Green, pil. terebinth. chio. gr. x. ter die.

1831, Jan. 12th. Discharge continues, though in less quantity, with scalding. Pergat.

19th. The discharge has ceased.

Presented well.

CASE 2.—*Gonorrhœa from the Vagina.* Mary Collins, æt. 15, admitted into Magdalen's Ward, under Mr. Green, Nov. 25th, 1830. Has had gonorrhœa for three months which is now rather profuse, but, as generally happens in the female, is productive of but little pain. Ordered to inject the decoction of tormentilla twice in the day.

Dec. 1st. Discharge somewhat less. A bubo has formed in the groin, tender and painful, but without discoloration of the integuments. The lotio plumbi to be applied to it. Injection continued.

9th. Gradual diminution of the discharge. Patient has piles, which are now enlarged and painful. Ol. ricini. Pergat.

17th. Discharge is at an end, and the enlargement in the groin has quite subsided.

23d. Presented.

CASE 3.—*Gonorrhœa, combined with Warts on Glans Penis.* James Drind, æt. 25, a patient of Job's Ward, Dec. 9th, 1830. He has six or seven small and smooth warts on the front and upper part of the glans, with one or two upon the prepuce; also a scanty gonorrhœa, which has continued for nearly two months: neither sores nor enlargement in the groin—warts not painful. It is about six weeks, he says, since they first appeared, and they have gradually acquired their present size, which is that of common shot. His bowels are open, and he has no other cause for complaint.

Ordered, mist. copaibæ, ℥jss. ter die. The muriate of antimony to be applied daily to the warts.

Dec. 16th. Warts much less—discharge has nearly terminated.

23d. Gonorrhœa cured. The warts are now destroyed to a level with the surface of the glans, and the vestiges of their bases are alone perceptible. The man quitted on this day.

CASE 4.—*Gonorrhœa combined with Warts.* Wm. Slater, æt. 20, admitted, Jan. 6, 1831, into Job's Ward. There has existed for seven days a gonorrhœa, which is now profuse. On the inner surface of the prepuce and under part of glans, near to frænum, several rough warts are placed of three months' duration. There is also considerable swelling of the foreskin, with redness. No glandular swelling in the groin—no ulceration on the penis. Mr. Green directed the warts to be removed by the scissors, and the sulphate of zinc lotion to be applied around the extremity of the penis.

Mist. copaib. ℥jss. ter quotidie.

Jan. 12th. Some of the warts on the under surface of the glans have been cut off, and the quantity of urethral discharge is diminishing.

27th. The greater number of the excrescences have been snipped off, and the others have been got rid of by the butter of antimony.

Discharge removed.

He left the hospital at this time.

CASE 5.—*Gonorrhœa, Warts, and Swelling of Knee-joint.* Nov. 25th, 1830. Elizabeth Walsh, æt. 20, admitted this day, having a sparing gonorrhœal discharge from the vagina, and some small warty excrescences on the outer side of the left labium pudendi, but no ulcers nor glandular swelling. She has not menstruated for three months, and there are pains of head in consequence.

Ordered to have liq. aluminis comp. as an injection, and to take mist. sennæ co. p. r. n.

Nov. 29th. The right knee-joint is attacked with pain and puffy swelling in front; the joint is generally enlarged and hot, and pain is increased on the slightest motion. An attack similar to the present articular affection was experienced by this patient about three weeks ago.

Hirudines xx. genû, and spirit lotion.

Dec. 1st. Pain and swelling of knee rather less, but heat and tenderness continue. Discharge from vagina very perceptibly diminished. Sleeps none at night. Lotion continued.

4th. Knee-joint somewhat less swollen, but the pain persists. No heat of surface. Cat. panis.

6th. Much relief has been derived from the poultice, and the swelling about the knee has a good deal declined. Same remedies.

10th. Knee easy, and swelling has much abated. She has pains in other joints of the body, and is very restless at night.

Mr. Green prescribed cal. gr. j. Pulv. ipec. co. gr. iij. o. n. Fever diet.

14th. Pains nearly gone. Some feverishness has been present, but the surface of the body is now cool, and the knee-joint is free from pain or other symptom of disease.

Omittr. cal. et pulv. ipec. co. House diet resumed.

21st. Experiences pain from the warts, which are touched with the muriate of antimony. No gonorrhœal discharge.

31st. Warts less.

1831, Jan. 6th. No remains of the warts.

13th. Presented.

The application of muriate of antimony seldom fails in destroying warts on any part of the penis, or female organs of generation, that have not attained an unusual magnitude and degree of hardness.

### II. Inflammation of Testicle.

*Case* 6. W. Bryant, æt. 30, admitted into Isaac's Ward, under Mr. Green, Nov. 11th, 1830. Three weeks ago a gonorrhœal discharge commenced from the urethra, and upon this there quickly supervened inflammatory swelling of the left testicle, he not refraining from active exertions when the discharge had appeared. He states that the urethral flux was not affected in any way by the swollen state of the testicle. No internal remedies have been used, with the exception of a dose of salts, nor any local applications. The testis is enlarged nearly to the size of the fist, being greater below than above. It feels firm to the touch, and somewhat hard, especially at the back and upper part, including the epididymis; there is not much tenderness; the spermatic chord is considerably enlarged, indurated, and tender, this state extending to within the inguinal canal. There is a good deal of external redness and heat, and the scrotum covering the enlarged gland is tense. He has no pain in the loins, and none of importance now exists in the organ itself. There yet remains some discharge from the urethra, thin and not having much the appearance of pus. No constitutional disturbance. Bowels regular — tongue clean and moist—pulse 64, small and feeble. The man is tall—about 6 feet, 2 inches.

Nov. 12th. Was visited by Mr. Green, who ordered—

Cal. gr. j. Ant. tart. gr. ¼. Pulv. opii, gr. j.—M. ft. pil. o. n. sumenda. Mist. sennæ c. cochl. iij. quotidie. Hirud. xv. Catapl. panis.

13th. Swelling, pain and tenderness diminished in the testicle. He has slept well, and his bowels are freely opened by the medicine.

15th. Testicle more soft—scrotum free from tension, and a great deal of redness—little pain on pressure—chord also less, and deprived in a great degree of its indurated feel.

17th. Still becoming less—no hardness, except in epididymis and back part of testicle, and also in the spermatic chord. The latter is, however, reduced in size, and not tender under pressure. The discharge from urethra has increased, which may be regarded as a counter-irritant.

20th. Mouth not yet affected by the mercury, but the swelling and hardness are gradually decreasing.

23d. Soreness of the gums since yesterday. Testis reduced to nearly its natural size; and the chord, though much less, feels hard, but is not tender. Mr. Green ordered the calomel to be omitted; and, as the gonorrhœal discharge was copious (having very perceptibly become more and more profuse) pil. tereb. chio, gr. x. ter die. The discharge was allowed to proceed hitherto, as it was considered likely to act by counter-irritation upon the testicle. Mercurial ointment, spread on lint to be applied over the testis.

25th. The gland has become of nearly its natural size, and the hardness at the upper part and epididymis continues to decrease.

At the patient's particular desire he this day left the hospital, a few days earlier than he would have been regularly discharged, since all disease was not completely removed.

*Case* 7. Hugh M'Cleod, æt. 22, taken into Job's Ward, Dec. 23d, 1830. Inflammation has taken place in the left testicle since the 20th, and it is now enlarged to twice its natural size, being hard and tender to touch. The surface of the scrotum investing it is red and heated—there is a very slight thickening of the spermatic chord—he has some lumbar pains, but very little in the gland itself. Pulse is full and hard, 100—tongue white—some nausea and thirst, with loss of appetite—belly costive. Gonorrhœa, which began about a fortnight since, now continues, but the discharge has very manifestly decreased since the testis has been affected; it is thick and purulent, though slight in quantity. When seen by Mr. Green on the 24th, he was ordered V. S. ad ℥xvj. and the following twice a day in the form of pill. ℞. Cal. gr. j. Ant. tart. gr. ¼. Pulv. opii, gr. ¼. M. Hirud. xij. Cat. panis. Fever diet.

Dec. 28th. Pulse still full and active pain increased in testis, and also in the loins—no change, with the exception of less redness of the scrotum, is to be observed in the inflamed organ. His bowels are costive, and the tongue white and furred.

V. S. ad ℥xij. Capiat pil. ter die. Mist. senn. c. p. r. n.

29th. The pain in loins being aggravated, blood was taken from that part by cupping, and he has found relief from the operation. The blood drawn on the first occasion had a slight buffy coat, but of this there are no traces in the present vessel.

31st. No pain of loins or testicle. The latter is much reduced in bulk, and there is less tenderness. The gums are slightly sore. Calomel omitted.

1831, Jan. 5. Testis nearly of natural size; it is free from tenderness and heat, and no induration can be felt either there or in the spermatic chord.

8th. The part to be covered with empl. saponis. Gonorrhœal discharge is still rather profuse.

10th. Pil. tereb. chio. gr. x. ter die.

19th. Discharge has nearly terminated, and what remains is thin and watery.

Was to-day presented, but ordered to continue the turpentine some little time longer.

III. Simple Ophthalmia and Rheumatism, connected with Gonorrhœa.

*Case* 8. William Green, æt. 63, admitted into the Eye Ward of Saint Thomas's Hospital, Jan. 13th, 1831, under the care of Mr. Green. There is slight ophthalmia of both eyes, accompanied by very indistinct vision, pain in the orbits and brows, and a dilated and sluggish pupil. He is unable to assign any direct cause for this attack, but when we mention he has had gonorrhœa for the last four weeks, it may perhaps be attributed to this source. The discharge from the urethra continues, and although purulent, is unattended by any local inflammatory symptoms; there is, however, some pain in the loins. No purulent matter is yielded by the conjunctiva, the surface of which indeed is scantily moistened with tears; the membrane itself is not thickened from vascular turgescence, and the cornea is clear in each organ. His pulse is 120, hard and full—the tongue white and furred, and he is thirsty. Mr. G. ordered cupping from the nape of the neck to ℥xij. A shade to be worn. Poppy fomentation. Cal. gr. ij. Ant. tart. gr. ⅙. t. d.

Jan. 16th. Pulse 110, softer. Inflammation of conjunctiva is considerably abated, and, together with the pupils having become more active, sight has improved. The gums are slightly tender, and he is rather feverish from this cause. Calomel to be taken at night only.

21st. Complains of pains, apparently rheumatic, situated in the joints of the upper extremities, and in the loins. The vision has increased, and the inflammation of the conjunctiva is subsiding. Pulse not augmented in fulness, nor frequency, but is rather hard. Ordered, cupping from the loins to ℥xvij. Hydr. c. cretâ, gr. iij. Pulv. ipec. c. gr. iij. M. t. d. Milk diet.

Jan. 25th. The discharge from the penis ceased immediately after the cupping in the lumbar region, in which situation the pain is also much less. His rheumatic pains in the limbs continue, though not so severe. Sight is perfectly good, and the eyes appear only what is termed "weak." He rests ill at night, and the pains of limbs become aggravated so soon as the surface is warm. To rub the linim. ammon. into the arms and legs twice a day.

27th. Pains of limbs are somewhat diminished, and he slept better last night. Pulse 100, not quite soft, but small. Bowels open.

1st Feb. Has less pain, and his pulse is at 96, soft—gums tender. Pil. o. n. Extr. sars. ʒj. Ex. decoct. sarsæ, ℥viij. bis die.

5th. There is slight ophthalmia from cold, but the pains of limbs have ceased. The gonorrhœal flow from the penis has returned.

6th. Ophthalmia does not exist today; the urethral discharge is increasing.

8th. Discharge from urethra more copious, and the rheumatic pains have almost entirely vanished. Pulv. cubebæ, ʒj. Ex. decoct. sars. ℥iv. t. d.

14th. No discharge from the penis, and no pain exists in the loins or extremities. He continues his medicine.

22d. The gonorrhœal secretion has once recurred since last account, but is now in very small quantity.

Was to-day presented, and is to continue the cubebs for a week.

Two cases of purulent gonorrhœal ophthalmia are detailed in the Fasciculus of this Journal for March, 1829, taken from the practice of Mr. Travers and Mr. Green.

### IV. Primary Syphilis.

Case 9. *Chancre on Prepuce.* Daniel Morby, æt. 20, Job's Ward, Dec. 2d, 1830. Has a small circular sore on the upper part of the prepuce, where this becomes continuous with the skin of the dorsum penis, which first appeared three weeks ago in the form of a minute pimple, and this soon breaking with a little discharge of matter, the present ulcer was established, the characters of which are as follows. It is circular, as we have before said, and has a smooth and rather shining surface—has no elevated edges, and consequently does not appear deeper than from simple loss of integument by ulceration; there is trifling hardness at the base. A thin and scanty discharge takes place from the surface. The pain arising from it is very insignificant; what he does feel he describes as rather itching, or pungency. He has no gonorrhœal discharge, nor glandular enlargement in the groin. General health not affected. Bowels costive.

Ordered to rub in Unguent. hydr. ʒj. four nights in the week. Lot. nigra to the sore. Mist. sennæ co. p. r. n.

Dec. 12th. Chancre but little altered in appearance. It seems to have spread in a slight degree, and to have some raised edges more perceptible.

18th. No increase of size in the sore.

28th. Sore has healed, and hardness remains on the spot.

1831, Jan. 6th. He continues the mercurial frictions, but the mouth does not seem yet affected. Cicatrix no longer feels hardened.

8th. Has tenderness about the fauces and gums investing the molares teeth. There is, however, no turgidity nor sponginess. The friction omitted.

11th. Left the hospital free from disease.

Case 10.—*Chancres on Prepuce.* John Connell, æt. 19, came into the hospital on the 3d December, 1830, under Mr. Green, having two large chancres on the right side of the prepuce, which cannot be drawn back over the glans in consequence of incomplete phymosis, (the point of the glans can be seen in endeavouring to denude it,) which appears to have been congenital. The chancres originated nine weeks since, seeming to him in the first place nothing more than red and angry pimples; ulceration shortly set in, attended with considerable local pain, and has extended slowly, but progressively, to its present dimensions. A little space intervenes between the borders of the two. The one nearer the extremity of the penis is almost an inch in length, beginning on the upper part of the preputium, adjoining to the centre of this, taking a transverse direction to the under surface, where it terminates close to the median line, or raphè in this situation, and thus occupying one side alone of the penis, all encroachment upon the opposite being apparently singularly prevented. It is much narrower in width, which is about a quarter of an inch. The other, placed behind, but still on the same half of the fore-skin, is nearly circular in shape. Both have defined edges, and a dry yellowish surface, no granulations being visible. Their bases are not hardened. No swelling in the groins, nor discharge from urethra. Has hitherto applied black-wash and poultices.

Ordered to rub in mercurial ointment, as in the preceding case.

Dec. 10th. Sores rather spread, and their surface is foul, there being a more plentiful and thick discharge of matter. Though wanting the hard base, which is to be regarded as the most decisive characteristic of chancre, Mr. Green considered them to be venereal, or syphilitic, and that they would heal under the use of mercury. Lot. nigra.

14th. Some redness has existed around the ulcers, but it is no longer visible. No pain in the ulcers, whose appearance is not changed.

21st. Ulcers reduced in size and cleaner, though not yet having lost their yellowish surface. No effect upon the mouth.

28th. One of the sores has healed, and the other is nearly well. Continues the lotion and the rubbing in.

1831, Jan. 4. Both sores healed, and have left large cicatrices. Mouth not yet touched.

8th. Gums tender and spongy—secretion of saliva slightly increased. No induration in the situation of the recent chancres. Omit the frictions.

11th. Left the hospital this day.

CASE 11.—*Chancre on Labium Pudendi.* Jane Barn, æt. 21, admitted Nov. 18th, 1830. Has a circular ulcer on the right labium pudendi, the size of a silver penny, which first attracted her notice three weeks ago. Its circumference is well defined, and, as well as the sore itself, is in some measure elevated above the surrounding surface. The base is indurated; it is of a yellowish tinge in the centre, to appearance from the secretion, which is thin, and it here seems slightly excavated. The parts adjoining are inflamed and tender. There is no gonorrhœal discharge. Some enlargement of a gland in the groin, which feels hard, and was larger a short time since than at present. Constitution bears little marks of injury. Ordered by Mr. Green, ung. hyd. ʒj. to be rubbed in four nights in seven. Lot. nigra and cat. lini to the sore.

Nov. 22d. Rather more inflammation exists in the neighbourhood of sore, but this is not increasing. Patient's rest at night is good, she suffering very little pain from the chancre.

27th. Inflammation has subsided, and the ulcer looks well. She complains of pains in her limbs and joints from cold.

Dec. 2d. Sore healed, but the swelling and hardness in the part have not yet become dispersed.

Dec. 10th. Gums a little tender, and spongy. Some slight enlargement of the integument remains in the situation of the healed chancre. Ordered to wash up after to-morrow night's friction.

17th. Presented well.

CASE 12.—*Chancres on Prepuce, and Bubo.* Francis Whitford, æt. 18, a sailor, was admitted into Job's Ward, under Mr. Green, Nov. 4th, 1830. He has two chancres situated on the posterior surface of the prepuce to the left of the median line, one of which first presented itself in the form of a pimple or head, as he says, six weeks ago, and about a week after sexual intercourse.

They are situated close together, their edges nearly coming in contact, and are on a line with each other. That nearest the extremity of the prepuce is small, and much less than the other, being not larger in circumference than a pea. It appeared a fortnight later than the greater one; this exceeds a sixpence in size, has a distinctly circumscribed border, which is elevated, notched, or denticulated, and possesses surrounding hardness. A layer of yellowish white lymph is at the bottom of each, but they are in a very inconsiderable degree excavated. Two weeks from the present time a bubo formed in the groin on the same side, which soon broke and has left an ulcerated surface, approaching in general appearance and character to those of chancre, but it is deeper than a primary venereal sore, though situated more towards the medial line of the body than buboes usually are. This ulcer is rather circular, and has a margin elevated and abrupt. His constitution, he tells us, has suffered very materially from the irritation of the disease, and the pain he feels in the sores is, and has been considerable.

Nov. 6th. Was visited by Mr. Green, and no change having taken place under the use of poultices and the black lotion, he ordered Cal. gr. j. Ant. tart. gr. ¼. Opii, gr. j. o. n. Mist. senn. c. cochl. iij. quotidiè. Lotion and cat. lini continued. The above quantity of opium was prescribed in consequence of want of sleep at night, which is frequently the case in primary sores, even if there be no unusual appearances of inflammation locally.

12th. Ulcers have nearly the same aspect; that near the groin has become more painful, and there issues from it a profuse thin watery secretion. Capiat pil. bis die. The chloride of lime-wash in lieu of the lotio nigra, to the sore near the groin, which has rather a more foul surface.

14th. Sores cleaning, and displaying some minute florid granulations; at bottom of larger sore they are more conspicuous and tend to fill up the cavity.

16th. No effect of the mercury is perceptible in the gums. Ordered to use ung. hydr. ʒj. four nights out of seven, and to discontinue the calomel. Local applications as before.

22d. The smaller sore on penis has healed, and the other nearly so; that in the neighbourhood of the groin is likewise contracting, and the granulations of it are florid and coalescing; in its centre there is no longer any hollow. The mercury has yet produced no effect upon the mouth. A slight pimply eruption has made its appearance during the last week upon the chin and forehead; the tonsils have been enlarged and painful, but are now diminished. A small ulcerated surface which looks quite healthy and has no specific character, being quite superficial, is to be seen on the right amygdala.

27th. Larger sore on penis nearly cicatrized, and that in groin less since last report—throat still painful from enlargement of the tonsils—gums not affected.

Dec. 1st. The remaining sore is now quite healed, and that on the groin is healthy and very much diminished.—Some hardness is felt around the cicatrices.

7th. Sore left in the groin is reduced to the size of a silver penny. He has a slight coppery taste in the mouth, with tenderness of the gums in front of the lower jaw.

14th. Sore well, and the gums are in some degree turgid and spongy.

21st. Gums sore—no salivation.—Swelling of tonsils has completely subsided, and he is able to swallow freely. The eruption which was noticed on the 22d has rather increased, and is of the nature of acne; it is seated principally on the face, and is also on the arms and chest. Mercurial friction to be now omitted. To take decoction of sarsaparilla, a pint, three times a day.

Dec. 30th. Left the house well.

CASE 13.—*Chancres and Bubo.* Daniel Hennesey, æt. 18, admitted under Mr. Green, February 3d, 1831. A foul circular ulcer exists in the situation of the frænum, the size of a silver penny, and from this towards the apex of the glans proceeds a line of ulceration, of the same foul character, terminating in a larger and more roundish form. These ulcers are of three weeks' duration; their surface is of a blackish colour, and afford an abundant and thin secretion. Very little pain is present. A great and hard bubo has taken place within the last fortnight on the right side. No discharge from the urethra. Ordered unguent. hydr. ʒj. four times a week. Mist. sen. c. p. r. n.

Cat. lini. Lotio nigra.

Feb. 14th. Sores have rather increased in dimensions, but their surfaces are cleaning, and already exhibit some granulations.

22d. A clean granulating surface is now displayed, from which a copious discharge of pus flows. Lotion alone used.

March 1st. Ulcers quite florid, but have not diminished. Gums not affected.

12th. Sores healing gradually—that upon the glans still communicates with the one upon the frænum and internal surface of prepuce by the same line, which is proportionally contracting.

22d. Ulceration only remains behind the glans. No mercurial effect yet manifest.

April 5th. Chancres have been healed for some time, but the cicatrices till now have been hardened, and the inunction therefore continued. For the last ten days some affection of the gums has become more and more manifest. He is now ordered to relinquish mercury.

7th. Presented well.

A kind of chancre, precisely similar to the foregoing, we will again relate in

CASE 14.—*Chancres and Bubo.* Michael Doyle, æt. 25, received into Job's Ward, Feb. 3d, 1831. There is a circular and unclean sore upon the frænum, having some hardness at its base, which commenced about a month ago, and has been slowly increasing. Upon the glans also there is a narrow streak of ulceration, proceeding from the same sore towards the apex. A bubo has existed in the right groin for a fortnight, which is hard, and not fluctuating, nor discoloured. Ordered Cat. lini. Lot. nigra.

Feb. 14th. Some diminution is noticeable in the sores, and they have become cleaner. Bubo is suppurating, and the swelling more prominent.

18th. Bubo has been laid open by a free incision, and the sores on the penis have healed. To rub in ung. hydr. ʒj. as often as in the preceding cases.

26th. Ulcer in the groin cleaning, and there is a profuse discharge of brownish pus from it.

March 4th. Healthy granulations are springing up from the bottom of the ulcer, which is hollow, and the edges overhang, but no burrowing of matter has occurred.

12th. Edges of the ulcer are less inverted, and the hollow space is nearly filled up. Hardness of the cicatrices on the penis has been perceptible, but seems declining.

22d. Ulcer considerably reduced and clean—no induration in the cicatrices. Rubbing in now left off. Black-wash to the groin.

April 7th. The edges of the inguinal ulcer have been pared, and this has now cicatrized. Was to-day dismissed, well.

CASE 15.—*Chancre under Fore-skin—Bubo.* John Harewood, æt. 20, Job's Ward, admitted December 16th, 1830. When the fore-skin is reflected from the glans penis, a sore is found to exist, which cannot however be completely exposed from partial phymosis, the prepuce being swollen, and rather red. The part of the sore that is visible has a clean and even surface, very little discharge is afforded by it, and in taking up the fore-skin in this situation between the finger and thumb, very considerable hardness is felt for some distance around it. A good sized bubo has formed on the left side, and the integuments over it are hot and discoloured. He first perceived the sore a fortnight ago, and the bubo three days afterwards. There is no discharge from the urethra. Ordered ung. hydr. ʒj. four nights out of seven. Hirud. xij. to the groin. Cat. lini, and the black-wash to the chancre.

Dec. 23d. Less swelling of the prepuce, and less pain from the sore.

31st. No phymosis now exists, all swelling having subsided. A sore is exposed on the inner surface of the prepuce, near the corona, in a healing condition; its surface is rather prone to bleed when the dressing of lint and black-wash is removed, and it is surrounded by hardness at its base—in short, it has the character of a syphilitic ulcer. Bubo greatly subsided, and no longer inflamed; it feels hard and firm to the finger, but is not tender.

Empl. ammoniaci, c. hydr. to be applied to it.

1831, Jan. 8th. Bubo nearly removed. Pergat.

14th. Mouth sore, sponginess and slight ulceration of the gums being manifest. The swelling in the groin is more dispersed—chancre cicatrized, and no hardness remains.

19th. No swelling exists in the groin. He omitted the frictions on the 15th.

Presented this day.

### V. SLOUGHING CHANCRES.

CASE 16.—*Sloughing Chancre, accompanied by Retention of Urine.* Thomas Cider, æt. 32, applied at the hospital Nov. 15th, 1830, late in the evening, suffering great pain from retention of urine. He stated that he had not passed any urine by the penis for more than 24 hours, but there was evidently no great deal contained in the bladder, as there was a want of swelling and distention above the pubes. The man was pallid and faint, and the surface was rather cold and clammy. A narrow slough, an inch in length, was hanging from the extremity of the penis, but as phymosis existed, with a swoln and red state of prepuce, its attachment and source, or the state of the glans, could not be seen. There was a pretty free discharge from the penis, of a purulent kind, and it was soon ascertained that he had applied here for admission on the preceding Thursday, with a chancre at the orifice of the urethra, and also another on the corona. A catheter was introduced into the bladder without difficulty, or causing unusual pain, and rather a large quantity of water was drawn off, perhaps a pint and a half. He was then ordered to bed and a poultice to the penis.

16th. Slept for some hours, and has made water twice without impediment. End of penis somewhat less inflamed, and there is a free discharge from it. Pulse 100, rather sharp—tongue a little white. A dose of house-physic was given to open the bowels.

18th. Has been quite free from pain, and made water by his own efforts easily—pulse 90—bowels open.

21st. The slough separated to-day; the inflammation is less, and the discharge very profuse. There is still sufficient swelling of the prepuce to prevent exposure of the glans.

26th. Has gone on well, but the phymosis and purulent discharge are unrelieved.

29th. Prepuce can now be drawn back, and a hollow ulcerated surface is presented to view, which occupies one side of the glans, from the corona to near the extremity of the latter, and from which the slough had separated. Its edges are irregular and rather foul, but it is clean in the centre, and a copious purulent discharge is given off from it. Black-wash applied to it, with cat. lini.

Dec. 2d. The ulcer on the glans has now somewhat of a circular shape, having lost the sloughy edges, and these are easily defined. The sore itself is contracting in size daily; its surface is covered with florid and healthy granulations, and good pus is discharged from them. Patient's health has not in the least given way, and he is at present upon the house-diet. No mercury has been given.

7th. Sore continues healing fast. Poultice omitted—black lotion on lint.

12th. Sore quite healed, and no induration is to be felt in its situation, except that from an ordinary cicatrix.

16th. Presented well.

CASE 17.—*Sloughing Chancre, destroying the whole Penis.* Charles Fardrell, æt. 30, admitted late at night on Tuesday, Dec. 7th, 1830. The glans and greater part of penis, to within an inch of its root, are destroyed by sloughing, being black and much diminished in size, and appearing as if retracted within the integument of the lower part of penis, which is nearly preserved entire. This is red, and its extremity (which forms that portion of the prepuce) being destroyed, the termination of it is irregular and truncated, and the edges of it are also sloughing. There is a very slight discharge from between the dead and sound parts of a purulent kind, and no line of separation is distinctly discernible. His urine now passes freely and without pain, and makes its appearance externally in the centre of the destroyed glans, that is, behind the natural situation of the opening of the urethra. The face is very red and hot; the patient has been accustomed to drink hard, but not much of spirits. Pulse full and hard, 120—tongue white and dry—bowels, he says, are open. He is at present suffering severe pain, but this has been more intense than now. We learn from him, that, three weeks ago, a sore formed under the foreskin, small and circular, which rapidly increased in the space of a week, to the size of a sixpence; phymosis then ensued, with redness of the parts. He has no doubt neglected himself, and the sore spreading whilst thus confined by the foreskin, sloughing commenced, on Friday evening last, in the upper part of the prepuce, without any further cause that he can assign, and has proceeded upwards to its present extent. On Saturday morning, he describes the end of the prepuce as "breaking," and a profuse thick and black offensive matter escaped. Sixteen ounces of blood were taken from the arm immediately; the part carefully supported, and a poultice applied, with lot. chlorid. calcis.

Tinct. opii, ♏xl. two hours later.

Dec. 8th. Very little sleep obtained in the night—face flushed, and of a dark red colour—pain has been very great—sloughing rather increased, no limit to be observed—blood buffed and cupped—pulse 120, not so full nor so hard. Was seen by Mr. South, who directed xxx. minims of tinctura opii at night; the chloride wash to be continued, and a stale beer ground poultice used.

9th. Sloughing somewhat extended, and no abatement of pain—he got little or no sleep in the night from the opiate—pulse and tongue as yesterday—at no period has there been much heat of skin, and the surface is now temperate.

Tinct. opii, ♏xx. at night—local remedies the same.

10th. The dorsum of the penis is destroyed as far as half an inch from the root—the sound skin is less red, and the sloughs appear inclined to separate from its edges—pain rather less, and he obtained a little sleep last night—face not so red and heated—pulse 120, sharp and hard—bowels open.

Mr. Green prescribed Pulv. ipec. co. gr. iij. Mist. pot. citr. ℥jss. 6tâ q. h. Fever diet, with beef-tea.

11th. Pulse less full and not so hard, 108—skin moist. The extent of sloughing is now defined, and the dead portion seems likely soon to come away—tongue clean and moist. He takes his beef-tea, and has not been so restless.

12th. Slough nearly separated. Patient is more desponding, and the pulse is rather hard and more frequent.

13th. Pulse 120. Urine has passed hitherto without pain or obstruction; it is at present voided with equal facility from below the sloughy portions, which are more nearly cast off.

14th. The parts have separated, and a tolerably clean surface is left. The urine is passed freely from the opening of the urethra, which is situated about half an inch from the root of the penis—there is no thirst—the pulse 120, more feeble.

Mr. G. prescribed Ammon. carbon. gr. v. Tinct. opii, ♏v. Mist. camph. ℥jss. 6tis horis. Port wine, ℥ij. daily. Sago and syrup.

17th. Surface cleaning and granulations are visible. No further loss of substance has taken place since the separation of the sloughs—pulse 120, fuller, and possesses some sharpness—tongue white and rather dry—skin moist, but warmer than natural—face more flushed on one side. He has rested better of late, and his bowels are open. Beef-tea and former remedies.

21st. Pulse 120, stronger—surface of wound cleaner than at any former period, but very painful when exposed—

patient sleeps tolerably well. Has been taking opii, gr. j. ter die, since the 18th, and has arrow-root allowed in addition to the other nutriment.

24th. At the upper part of the wound there has formed a small slough, which will shortly separate. The ulcerated surface looks healthy, and yields a copious discharge. The remaining portion of the penis has contracted considerably in size. The patient's general condition is favourable, as before.

28th. Farther contraction has taken place in the sound portion of the penis, and this has rather a shrivelled appearance. Some part of the slough, at the upper part of the wound, has been discharged amidst the very profuse secretion of pus; and the extent of the wound itself is a good deal reduced, its surface below the sloughing point being quite healthy. The urine flows without impediment through the aperture of the urethra in the centre of the sore, and without pain—strength of the patient is rather diminished, but his face is still red, though not flushed, and the pulse is at 110, though smaller and more feeble—tongue clean and moist—he takes beef-tea and milk—is much depressed in spirits.

Mr. Green ordered Quin. sulph. gr. ij. Inf. ros. comp. ℥jss. t. d. Continue the poultice and chloride.

31st. All the sloughs, from the circumference of the wound near the pubes, are thrown off, and the wound itself is decreasing. A very plentiful discharge continues. Ordered opii, gr. ij. o. n., as he has latterly obtained scarcely any sleep, and the draught, with Dover's powder three times a day, to be omitted. Calomel and lime-water substituted for the chloride of lime. The opium pill thrice daily is of course to be taken no longer. Porter, ℔j. daily.

1831, Jan. 7th. Wound has gradually contracted, together with the remaining part of the penis. A small slough has recently formed in its centre—pulse still 110, rather feeble.

11th. The small slough has been detached; contraction of the parts is progressive, and the surface of the wound healthy. Pulse is unaltered; the tongue moist and tolerably clean. Continues the medicine, wine, beef-tea, &c.

14th. Wound gradually diminishing, and cicatrizes most favourably. His rest at night is very bad, and the appetite delicate. Loathes his beef-tea, but can take a mutton-chop. Mr. Green directed the opium to be discontinued at night, as he thought the patient might probably be able to sleep better without it, having been so long continued; and his porter to be taken with his supper.

℞. Inf. calumb. ℥jss. Conf. arom. ℈j. Tinct. calumbæ, ʒj. M. ter die.

18th. Complains more of the want of sleep, but in other respects he is progressively getting better. Ordered to resume the opium at night.

20th. Bronchitis came on last night, accompanied by very abundant expectoration. The pulse was very rapid and feeble; he was visited by one of the apothecary's apprentices, who was fearful of using the lancet in the present condition of the patient, and prescribed a blister to the chest, and exhibited antimony in moderately large doses. The patient is now quite free from pain, and has experienced less difficulty of breathing since the blister was applied. Cough is only present when the mucus requires to be got rid of, and it is not painful—pulse 140, weak—the expectoration seems to collect about the bronchia, and the patient unable to throw it up, from debility.

Jan. 21st. Pulse 140, irregular, and very feeble. Bronchitis has increased, and the man is much weaker. Dr. Elliotson saw him about three o'clock this afternoon, and confessed himself quite at a loss how to act, seeing, on the one hand, that the disease, if allowed to proceed uncontrolled, would destroy life, and, on the other, that there was not strength remaining in the system, to bear the means necessary to subdue the inflammation going on within the chest. He, however, ordered bleeding ad deliquium; three grains of opium to be given immediately afterwards, and five grains of calomel every three hours. Forty ounces of blood were drawn, though, unfortunately, not in so large a stream as could be wished, and then true syncope was not induced, although the man was sitting up in bed. After the bleeding the poor fellow felt confident he should soon get better, but his powers gradually failed, and he died, without pain and retaining perfect consciousness to the last, at half-past three o'clock on the following morning.

An examination of the body was not allowed by the friends of the deceased.

This severe pulmonic attack was unexpected, but from the first few hours a fatal termination was anticipated in the enfeebled condition of the patient's mind and body. Nothing, however, could be more successful than the detachment of the sloughs from the genitals, and the ulcerated surface at the time of death was perfectly clean and cicatrizing.

It will be seen that mercury was not employed in either of these two cases! In consequence of the rapid destruction of substance it is supposed that absorption of the poison of lues seldom takes place in the sloughing chancre, and certainly secondary symptoms following this sore are uncommon, so rare indeed as to warrant us in not resorting to this mineral until they do appear, which after all will be the fittest period for the interposition of the remedy.

### VI. Combination of Gonorrhœa and Syphilis.

CASE 18.—*Gonorrhœa — Chancres— Phymosis.* Nov. 25, 1830. John Bloye, æt. 19. We observe phymosis, and great swelling of the prepuce, which is slightly reddened, and the integuments are so much enlarged from effusion of serum that it is impossible to discover whether any chancres exist beneath the foreskin by external examination. He has been unable to expose the glans for nearly a fortnight. There is a profuse gonorrhœal discharge of three weeks' standing. No external sore, and the glands in the groin are not enlarged. Ordered — to inject the lot. plumbi around the glans under the foreskin— pil. tereb. chio. gr. x. b. d.

29th. Inflammation of the prepuce less, and the discharge also diminished —has slight superficial ulceration on right tonsil, rather circular, but still devoid of specific character, for which a gargle of chloride of soda is directed. —Pergat.

Dec. 3d. The ulcer in the throat is healing, and less soreness is complained of by the patient. Phymosis not so painful, and the enlargement is a good deal reduced.

6th. Swelling at the end of penis has abated considerably, but the glans cannot be denuded. At the pointed extremity of the prepuce there is considerable hardness, and also near the frænum on the left side of the penis, a circumscribed induration is to be felt, in both as if from chancres. Ordered to rub in unguent. hydr. ʒj. four nights in the week.

14th. Hardness on left side of penis more distinct, in consequence of the inflammation of the prepuce disappearing, but this part cannot yet be drawn over the glans penis.

21st. No discharge from urethra now remains. The end of the glans can be exposed, and some ulceration is observable on the inner surface of the foreskin when this is retracted, but it is superficial and not in the situation of the hardened spots formerly spoken of, where true chancres still apparently exist. No influence of the mercury is yet evinced in the mouth. To use ung. hydr. ʒiss.

28th. No change worthy of being mentioned is effected in the condition of the penis, nor is there any mercurial action yet noticeable in the system.

1831, Jan. 8th. Patient is still unable to retract the prepuce; no swelling exists in this part, nor redness. The hardness so characteristic of chancres is undiminished in every direction under the foreskin.

14th. No alteration is to be observed in the state of the penis, and the mouth is not affected by the mercury.

16th. Some tenderness of the gums and back part of the jaws is to day felt, and the hardness in the situation of the chancres is less perceptible, but he is unable to uncover more of the glans.

27th. The induration is much less distinguishable, but some adhesions appear to have formed between the glans and prepuce, as there is no motion between them.

Feb. 1st. Hardness has become gradually dispersed, and it remains only in the trifling degree that a common cicatrix would possess. Ordered to wash up.

3d. Presented well.

CASE 19.—*Gonorrhœa, accompanied by Inflammatory Phymosis — Chancres.* Edward Dunn, æt. 18, made a patient under Mr. Green in Job's ward, Jan. 6th, 1831. The extremity of the penis is considerably enlarged from swelling of the glans and prepuce alone, as the body of the organ is of natural size and not involved in the inflammation. Great redness is observed in the swollen portion, which is hot and excessively painful. There is complete phymosis. The present inflammation has existed for nearly a fortnight; clap commenced about five weeks ago, and the discharge is now copious. He denies that any ulceration has taken place on the inner surface of the prepuce, or on any part of glans penis. A bubo is situated in the right groin, but is not larger than a walnut, hard, and not inflamed. Mr. Green ordered—mist. sennæ, co. cochl. iij. quotidie. Cat. panis, and lot. plumbi to the penis.

Jan. 12th. Inflammation and swelling are considerably diminished. The redness is also of a less vivid hue.

18th. Has had an attack of pleuritis, which has been treated by venesection, blistering and antimonials. The disease is now subdued. Inflammation of the penis considerably abated, together with diminution of the gonorrhœal discharge.

25th. The swollen and inflamed state of the end of the penis has become so much reduced that a circumscribed hardness is distinguishable between the frænum and apex of the glans, produced by a chancre. On the outer surface of the prepuce also some ulceration has taken place on the anterior part, which has an indurated base, but not plainly defined edges. Ung. hyd. ʒj. to be used four times a week.

31st. Hardness is still more distinct near the frænum, now that the swelling is further diminished. The external ulceration possesses a more specific character than hitherto, and has spread. We are now told by the patient that he did notice a sore on the glans before the phymosis occurred; this latter now continues.

Feb. 5th. Sore, externally situated, has put on a clean and granulating surface, and is also less.

15th. The internal hardness is much diminished, and the ulcer has been progressively contracting. No effect yet upon the mouth.

22d. Ulcer perfectly healed; the hardness beneath the foreskin has also vanished, and phymosis no longer remains.

March 11th. Mouth sore, and the friction is now omitted. Decoct. sarsæ co. ℥vj. b. d.

17th. Discharged well.

CASE 20.—*Phymosis from Chancres—Gonorrhœa — Suppuration in Integuments of Penis, and in Corpus Spongiosum.* Charles Clark, æt. 30, Job's ward, Dec. 2, 1830. States that three weeks since a chancre was noticed on the glans penis, and in consequence of his neglecting himself the parts became inflamed, and phymosis ensued ten days ago. Subsequently to this event the inflammation has been progressive, and matter has collected beneath the cellular tissue of the penis, upon the dorsum, which latter is in consequence immensely swollen and red; fluctuation in the part is very distinct, but there is no particular prominence, or pointing. This, however, being punctured with a lancet, a considerable quantity of fetid pus escaped with a forcible jet, and the swelling was instantly very much reduced. The pain he has endured from this cause during the last week has been, of course, severe. Some pus escapes from the end of the penis. Slight glandular enlargement exists in the groins, but no discoloration of the skin, nor tenderness. Pulse is 100, not hard —tongue whitish, and dry in some degree. He complains of thirst, and does not sleep by night, at which time he perspires rather freely; but altogether the man's aspect bespeaks trifling constitutional excitement. Ordered—cat. lini. to the penis. House medicine occasionally.

Dec. 4th. Has been greatly relieved by the evacuation of the pus. The enlargement of the penis is less, and the redness is not so bright. The edges of the puncture look dark and unclean, as if disposed to the sloughing process—discharge from it is slight, and we do not notice any from the urethra, the orifice of which is not, however, visible. Cat. lini.

6th. Size of penis much reduced—edges of puncture are separated to some distance, but are clearly granulating—there is a free purulent discharge from the part, and the abscess appears to be closing. He rests tolerably well at night—his skin is temperate and moist, the tongue cleaner, and the feverish symptoms have become gradually less. Pergat.

9th. Tumefaction greatly subsided, and the redness and pain are now very insignificant.

14th. The swelling and inflammation have progressively diminished; very little purulent matter escapes from the puncture, which is clean.

21st. Patient has had rigors, followed by a hot and sweating stage, during the last three days, not occurring at regular intervals, and to-day they have been more severe than heretofore. The perspiration is very profuse. Pulse 100, of moderate strength, and not incompressible—skin, after the sweating stage has passed by, is dry and hot—tongue furred, and white—has vomited during the night—bowels costive—loathes every kind of food. Discharge of pus from the puncture has been very scanty, and the part has almost healed. There is no appearance of inflammation, or suppuration, about the genitals, and the abscess on the dorsum penis has contracted, so that the circumference of the penis is little larger than natural. Mr. Green prescribed Pulv. scammon. c. hyd. gr. xv. statim. Magnes. sulph. ʒj. Vin. ant. tart. ♏xx. Mist. menthæ, ℥iss. M. ter quotidiè. Poultice continued.

23d. The rigors have recurred each day, though slight. He now perspires very copiously, the skin being constantly wet, and rather warm. During the two last days the bowels have been much purged, and he has had frequent vomiting, but there is no tenderness of the abdomen evinced under pressure. The pulse is at 120, small and rather feeble, not hard—tongue furred and of a yellowish colour, but not dry. Does not complain of pain in abdomen, nor in any other part. Continues the medicine.

24th. Has experienced more severe pain since yesterday, seated in the bowels and augmented by pretty firm pressure — pulse is 120, small, but sharp. The vomiting and purging have continued, the pain being temporarily increased during evacuations and before them, and the man has had two or three fits of shivering this morning. His tongue has likewise become more foul. V. S. brachio ad ℥xvj. Hirud. xx. abdomini. Mist. potass. citr. eff. c. Succo limonis, 4tis horis.

25th. There is less pain of belly, and he has had no rigors—rested better last night—pulse 120, not so sharp. Two fluid motions have passed from the bowels without pain, and the vomiting has ceased—tongue is white, and more moist—tenderness of abdomen under pressure diminished considerably.—Pergat.

26th. No return of shivering, nor of abdominal pain—pulse 96, has lost its sharpness.

28th. The rigors have not since recurred, and there no longer exist any symptoms of inflammation within the abdomen.

30th. Patient continues easy, and his bowels are open without pain, and no tenderness remains.

31st. Pulse 80, soft and regular—tongue whitish. Inflammatory swelling of penis has greatly subsided.

Jan. 3d, 1831. Local inflammation of the penis has been progressively declining, but the prepuce and extremity are still so much swollen as to prevent the glans being denuded. Poultices are continued.

8th. There is no abatement of swelling, and there is a very profuse purulent discharge from the urethra, which was noticed in the first place, but has recently become more copious. No particular hardness is to be detected near the extremity of the penis, indicating the situation of chancre.—Pil. terebinthinæ chio. gr. x. ter quotidie.

18th. Matter has re-accumulated on the dorsum penis, near the former situation; a lancet being thrust in, about half an ounce of pus escaped. Cat. lini.

27th. There is a free discharge of matter from the opening, and the tumefaction has abated very considerably; but he is still unable to denude the glans—gonorrhœal discharge is rather diminished.—Pergat.

Feb. 1. Swelling of the penis has been gradually increasing within the last few days, and there is some developed also in the scrotum; these parts are now very greatly enlarged, tense, and the seat of throbbing pain and heat. There is much hardness of the corpus spongiosum, with a sense of distention imparted to the fingers upon examination, nearly from the commencement of this body to the glans, and suppuration has taken place in it. The man has been harassed with more feverishness, indicated by a moist and clammy skin, and often by a flushed cheek. Mr. Green made an incision into the spongy body, and let out an immense quantity of very offensive pus, to the great relief of the patient.

5th. A very plentiful discharge has flowed from the wound in the perinæum and the swelling both of penis and scrotum is perceptibly less.—Patient is free from febrile excitement, and has rested well.

8th. There is still less swelling, and the discharge is likewise decreasing. The incision presents some sloughs, which are merely superficial.

14th. A purulent discharge from the incision continues, and tolerably abundant. The parts have been gradually diminishing, and very little redness remains.

18th. A gradual abatement of swelling has taken place, and the discharge daily diminishes. — Phymosis remains, and a rather abundant discharge from the urethra. Poultices, &c. as before.

22d. The enlargement has progressively decreased, with the quantity of discharge from wound and from urethra.—Ardor urinæ has recurred.

March 1st. Wound made in the corpus spongiosum has almost healed, and no urine has ever escaped through it. The penis is not reduced in size, and the phymosis and gonorrhœal discharge are not relieved in any material degree. —Pergat.

8th. A circumscribed hardened spot exists in the corpus spongiosum at the point where the incision was made into it. There is a hard swelling in the site of the abscess on the dorsum penis, and the gonorrhœa persists, but is less severe.

17th. He was made an out-patient of the hospital to day by his own desire, the parts having further subsided, and the induration in the corpus spongiosum being somewhat removed.

The quantity of gonorrhœal matter is very trifling. He is directed to continue the poultices, and the chio turpentine.

We should imagine that the patient's constitution would not be secure from secondary symptoms unless mercury be administered. As there is very considerable thickening and enlargement of the prepuce, which yet firmly envelops the glans, venereal ulcers may exist beneath it, not to be detected by the fingers externally applied.

### VII. Superficial Sores and Local Inflammation.

*Case* 21. James Noin, æt. 35, admitted into Job's ward during Mr. Green's week, Nov. 29th, 1830, having several superficial sores on glans penis and lining fold of præputium, of four weeks' standing. The glans was of a deep red colour, the integumentary membrane appearing inflamed, and the inflammation confined to it alone. He had neither bubo, nor gonorrhœa. The sores were painful, and plainly in an irritable state. They were observed very speedily after connexion, within two days. He had made use of no local application either to protect the inflamed surface, or to endeavour to heal the sores, and consequently their appearance is the more angry. Mr. Green ordered the penis to be supported against the abdomen, and lot. spirit. to be applied around the extremity of the penis on linen.

Dec. 7th. Sores considerably healed, and there is less of redness and superficial inflammation in the glans and prepuce. Continue.

14th. A copious purulent discharge comes from the sores, which look kindly.

21st. Sores on penis nearly healed.

28th. The sores have perfectly cicatrized; there is no remaining hardness, and the inflammation has disappeared. He now left the hospital voluntarily.

CASE 22.—*Superficial Sores near Corona Glandis.* Richard Pavy, æt. 22, Job's ward, Dec. 28, 1830. There is a considerable degree of inflammation about the end of the penis, and the prepuce is red and swollen. Around the corona glandis several small and superficial ulcers exist, and some excoriation; from the former there is a free purulent discharge of a healthy character. He has no clap, nor enlargement of the glands in the groin. The present sores have been stationary, and appeared a fortnight ago, a day or two after intercourse. Ordered to apply lot. nigra.

Jan. 1st. 1831. Less inflammation in the prepuce, and the ulcers have not increased. Continue.

12th. Sores healing.

19th. The ulceration has completely healed, and the patient is presented well.

It would be difficult, nay, perhaps more than possible, for any one to say, from reading an account of such cases as we have just given, whether the sores were venereal, or not; or whether the exhibition of mercury was required. This important question must be decided, we apprehend, from present appearances alone, and we need not remark how carefully such must be weighed in our minds, with the particular history accompanying each individual case. In the two instances we have particularized, the sores had the aspect of ordinary ulceration, and its properties, namely, secretion of purulent matter. They were observed very soon after sexual indulgence, and had no external characters of chancre. Their speedy cicatrization under the use of simple applications must put their true nature beyond doubt, and on all occasions where uncertainty may justly exist, we think simple means should be tried, which, if failing in curing, or changing their appearance, should be superseded by the specific remedy.

### VIII. PARAPHYMOSIS.

*Case* 23. George Johnson, æt. 23, a sailor, was admitted on Thursday, the 23d December, 1830, into Job's ward. States that he first observed a gonorrhœal discharge from the urethra on Sunday last, in the evening of which day paraphymosis also occurred. The entire penis is excessively swollen and red, more especially the prepuce, which is firmly girt down at the corona glandis, where a slight stricture exists, and is distended by effusion of clear serum, the part possessing a considerable degree of transparency. Under this condition of the penis he has, of course, endured constant agony, and he is now labouring under great febrile excitement. There is prostration of strength, and the countenance is pallid and moist — the pulse sharp, and hard, 120 — tongue white and dry—thirst—bowels constipated — gonorrhœal flux is profuse; the urine is voided with some scalding, but with no impediment from the stricture at the corona.

On the following day the patient was visited by Mr. Green, when the parts were in the same condition as above described. The man had passed a sleepless night, as he had done previously to admission. Mr. Green at first tried to replace the prepuce by emptying the glans of its blood under pressure with the fingers of one hand, and by traction forwards of the integument behind this with the other; this plan, however, not succeeding, he made a small incision upon the dorsum of the penis with a lancet, and the foreskin being then forcibly drawn forwards and seized at the extremity was, after some little time, brought over the glans, strangulation having continued for five days. The means of reduction were of necessity painful, and a state bordering on syncope was thereby produced. Some ulceration was found to have taken place on the inner surface of the prepuce near to the point of the incision, from the great pressure the parts had undergone. Rags wet with lotio plumbi, cold, are to be applied around the penis, and the bowels to be opened by mist. sennæ comp.

Dec. 28th. Swelling and inflammation are greatly reduced; the surface of the incision and that of the ulcer are free from sloughing, and in a favourable state for granulation. Discharge from the urethra very copious. Pil. terebinth. chio. gr. x. ter die.

Jan. 1, 1831. Swollen state of penis has very much subsided, and it is no longer discoloured, nor painful—an extensive but superficial ulceration is produced on the inner and upper part of the thigh near the groin, which the patient attributes to the irritation of the moist linen applied to the penis. It is covered nearly all over with a black encrusted matter, and the surface, when this has separated in any part, appears underneath red and in an irritable condition. Cerat. calaminæ ordered.

10th. A gonorrhœal matter still flows from the urethra, and this is the only ailment the man has. The incrustations have been cast off from the thigh, and no ulceration exists there.

18th. The bladder has become irritable from the excessive stimulus of the turpentine—he passes his water nearly every ten minutes, and has a continual uneasiness in the hypogastric region, but has no tenderness there on pressure.

Pills to be intermitted—hirudines 15 to the sacrum, and a blister afterwards, which is to be removed so soon as vesication has taken place.

20th. Is no better, being obliged to void his urine three or four times in the course of an hour — has had chills, followed by sweating — pulse 100, not full, nor hard—skin temperate—tongue white, but moist — no thirst — bowels open—he has rested ill.

22d. Irritability of the bladder has ceased, and the febrile state attending it subsided in a great measure.

27th. Some discharge remains, with ardor urinæ, but excepting these he has nought to complain of.

Feb. 8th. A thin and colourless discharge continues from the urethra, but it is nothing more than gleet.

17th. Presented well.

CASE 24. — *Paraphymosis.* James Stevens, æt. 24, was admitted Jan. 13th, 1831, into Job's ward. Reports he has had paraphymosis for nine days, and noticed a discharge from the urethra about a week before it took place. This has now nearly terminated — very slight inflammation is present, and he endures no pain. Some ulceration has taken place in the constricted part of the prepuce, but there is very slight enlargement of the extremity of the penis. The constriction behind the glans is very firm, and as the patient would not make up his mind to suffer the pain from the replacement of the parts, Mr. Green declined making any attempt, which he was not at all certain would prove successful. No chancre would appear to have formed on the glans, or inner surface of the præputium—cat. panis, to be applied cold.

Jan. 16th. The surface of the glans has been dry, and the cuticle cast off in small pieces, but it is now in its natural moist state, and the swelling has subsided. The ulceration is clean, and the attachment of the prepuce seems to be on the right side only.

20th. The foreskin has come over the glans for a considerable distance without any assistance from art, but the adhesion is not broken through.

27th. The entire glans is now covered by the prepuce. A rather profuse purulent discharge issues from the ulceration on the outer surface, which has however a natural and healthy appearance.

Feb. 1. Ulceration is somewhat diminished in extent, but phymosis now exists.

5th. No ulceration remains, nor other ailment but phymosis.

17th. Presented well, but the glans cannot be perfectly denuded.

Had space permitted, we intended adding some cases of secondary syphilis, and some also of primary and secondary disease complicated in the same individuals. These, however, we find from the great length to which our present paper has extended, must be deferred with other surgical cases of interest to another occasion.

BETA.

